# Analysis of nanomedicine applications for inflammatory bowel disease: structural and temporal dynamics, research hotspots, and emerging trends

**DOI:** 10.3389/fphar.2024.1523052

**Published:** 2025-01-08

**Authors:** Hong-Yu Jiang, Bo Shao, Hong-Da Wang, Wen-Qi Zhao, Shao-Hua Ren, Yi-Ni Xu, Tong Liu, Cheng-Lu Sun, Yi-Yi Xiao, Yi-Cheng Li, Qiang Chen, Peng-Yu Zhao, Guang-Mei Yang, Xu Liu, Yu-Fan Ren, Hao Wang

**Affiliations:** ^1^ Department of General Surgery, Tianjin Medical University General Hospital, Tianjin, China; ^2^ Tianjin General Surgery Institute, Tianjin Medical University General Hospital, Tianjin, China; ^3^ Tianjin Key Laboratory of Precise Vascular Reconstruction and Organ Function Repair, Tianjin, China; ^4^ Department of General Surgery, The Affiliated Hospital of Inner Mongolia Medical University, Hohhot, China

**Keywords:** bibliometrics, nanomedicine, inflammatory bowel disease, research trends, emerging frontiers

## Abstract

**Background:**

The application of nanomedicine in inflammatory bowel disease (IBD) has gained significant attention in the recent years. As the field rapidly evolves, analyzing research trends and identifying research hotpots are essential for guiding future advancements, and a comprehensive bibliometric can provide valuable insights.

**Methods:**

The current research focused on publications from 2001 to 2024, and was sourced from the Web of Science Core Collection (WoSCC). CiteSpace and VOSviewer were employed to visualize authors, institutions, countries, co-cited references, and keywords, thereby mapping the intellectual structure and identifying emerging trends in the field.

**Results:**

The analysis covered 1,518 literature across 447 journals, authored by 9,334 researchers from 5,459 institutions and 287 countries/regions. The global publication numbers exhibited an upward trend, particularly in the last decade, with China leading as the top publishing country and the Chinese Academy of Sciences emerging as the foremost institution. Dr. Xiao Bo is the prominent figure in advanced drug delivery systems. This interdisciplinary field, which spans materials science, pharmacy, and medicine, has seen influential publications mainly concentrated on targeted nanoparticles treatment for IBD. Keyword analysis revealed that current research hotspots include drug delivery, immune cell regulation, antioxidant damage, intestinal microbiota homeostasis, and nanovesicles.

**Conclusion:**

This study offers a comprehensive overview of global research landscape, emphasizing the rapid growth and increasing complexity of this field. It identifies key research hotspots and trends, including efforts to enhance the precision, efficacy, and safety of nanomedicine applications. Emerging directions are highlighted as crucial for further progress in this evolving area.

## 1 Introduction

Inflammatory bowel disease (IBD), encompassing ulcerative colitis and Crohn’s disease, represents a group of chronic, idiopathic inflammatory conditions affecting the gastrointestinal tract (Abraham and Cho, 2009). The epidemiologic patterns of IBD are evolving, and at the turn of the 21st century, it has become a major public health challenge worldwide (Buie et al., 2023). The incidence of IBD varies greatly by geographical region, with rapidly increasing rates in newly industrialized countries (Asia, Eastern Europe, and Latin America), ranging from 10 to 80 per 100,000 individuals (Wang et al., 2023). While the incidence appears to be stabilizing in Western countries, the burden on healthcare systems remains high, with prevalence surpasses 0.3% (Ng et al., 2017; Kaplan, 2015). Particularly concerning is the steady rise in the incidence of pediatric IBD over time (Sykora et al., 2018). These trends suggest that the global impact of IBD is increasing and that the healthcare burden of managing this complex disease is substantial.

Patients with IBD often suffer from abdominal pain, diarrhea, rectal bleeding, and possible extraintestinal manifestations due to impaired intestinal mucosal barrier (Turner, 2009; Wehkamp et al., 2016). Diagnosing IBD based solely on a constellation of symptoms and signs can be challenging in clinical practice. Meanwhile, mainstream detection methods such as colonoscopy, capsule endoscopy, computed tomography (CT), and nuclear magnetic resonance imaging (MRI) (Plevris and Lees, 2022; Flynn and Eisenstein, 2019) are not only enormously distressing for patients but also insufficiently sensitive, especially at the early stage of IBD. Currently, there are no definitive interventions for IBD (Fu et al., 2023; Morris and Chu, 2015). Clinical medical management mainly relies on aminosalicylate, corticosteroids, and immunosuppressants (Agrawal et al., 2021; Wright et al., 2018). However, these treatments generally provide symptom relief rather than addressing the underlying causes of IBD. Long-term use of these medications can lead to serious adverse effects, including nausea, vomiting, headache, and liver and kidney toxicity (Stallmach et al., 2010; Rosen et al., 2015). Worst of all, patients with IBD face an elevated risk of developing life-threatening diseases such as colorectal cancer (Porter et al., 2021). Many patients eventually require surgical intervention, and in some cases, the condition can be fatal (Keller et al., 2019). These studies highlight the urgent need for research on IBD to facilitate the improvement of diagnostic sensitivity and the effectiveness of disease treatment.

Nanomedicine, which refers to the applications of nanotechnology in medicine (Kim et al., 2010), is revolutionizing the diagnosis and treatment of various diseases, including IBD, by leveraging the unique structure, as well as physical and chemical characteristics of nanomaterials (Patra et al., 2018; Hu et al., 2023). Firstly, nano-based imaging allows for the early detection of IBD and the monitoring of disease activity. Nanoparticles with different sizes and coatings can specifically accumulate in the colitis areas, providing enhanced visualization of inflamed regions and enabling more accurate and earlier diagnosis (Yue et al., 2023; Elinav and Peer, 2013). Nanomaterial-based probes that target the molecular or cellular processes involved in IBD pathology can detect subtle metabolic changes and inflammation more effectively than traditional methods, allowing for prompt disease assessment and timely treatment adjustments (Wu et al., 2016; Fu et al., 2023). Additionally, the use of nanomaterials in the treatment of IBD is an emerging and promising area of research, offering primary benefits such as improved drug delivery, enhanced stability, and targeted therapy (Zhang Y. et al., 2023). Nanomaterials can improve the chemical and physical properties of drugs, including solubility, stability, and circulating half-life. Besides, nanoparticles can be engineered to deliver drugs specifically to inflamed tissues, reducing systemic exposure and minimizing side effects (Liu et al., 2021; Naeem et al., 2020). This targeted delivery increases the concentration of drugs at the site of inflammation, improving therapeutic outcomes (Yang et al., 2024). Inspiringly, nanomedicine allows for the combination of diagnostic and therapeutic agents, enabling theranostic applications that provide real-time feedback on treatment efficacy (Patra et al., 2018; Zhang Y. et al., 2023; Yan et al., 2022). Naha et al. (2020) developed dextran coated cerium oxide nanoparticles (Dex-CeNP) as a CT contrast agent for IBD. Dex-CeNP not only accumulated in inflamed areas and protected against oxidative damage but are also efficiently cleared from the body within 24 h. This capability is particularly valuable in managing IBD, where monitoring disease progression and response to therapy is critical. In summary, nanomedicine offers significant advancements in the diagnosis and treatment of IBD. By harnessing the unique properties of nanomaterials, it is possible to develop more effective and targeted therapies that improve the outcomes of patients and reduce the burden on healthcare systems. Although the applications of nanomedicine in IBD have been explored from multiple-perspective, a comprehensive and systematic analysis of the field remains lacking.

Bibliometrics refers to an interdisciplinary science that utilizes mathematical and statistical measurement techniques to quantitatively analyze scholar publications (Moed, 2009; Yuan et al., 2023). The holistic and objective bibliometric evaluation of literature on a specific topic can enhance understanding of the current research landscape and assist in selecting research directions (Dong et al., 2023). As far as we are aware, there has not been any bibliometric analysis in this field to data. This study conducted a bibliometric visualization analysis of literature related to nanomedicine applications in IBD, including authors, institutions, countries/regions, journals, co-cited references, and keywords. The aim is to objectively assess the research structure and development trends in the major fields of IBD nanomedicine, offering new insights and clues for the subsequent study in this area.

## 2 Materials and methods

### 2.1 Data source and search strategy

The Web of Science Core Collection (WoSCC) bibliographic database stands as one of the largest and the most comprehensive electronic scientific literature databases globally (Jin et al., 2023), widely favored by academic researchers. An extensive search was conducted on the database on 15 July 2024, and relevant literature published was downloaded since 2001 for analysis. The search strategy utilized in this study was set as follows: TS = (“Inflammatory bowel diseases” OR “Ulcerative colitis” OR “colitis” OR “Crohn Disease”) AND (“nano*”). The type of publication was limited to Article or Review, and the language was set to English only. Subsequently, the search results were documented with the content of “Full Record and Cited Reference” in the “Plain Text” format. A total of 1,554 papers were included in the analysis.

### 2.2 Data analysis and visualization

**FIGURE 1:**
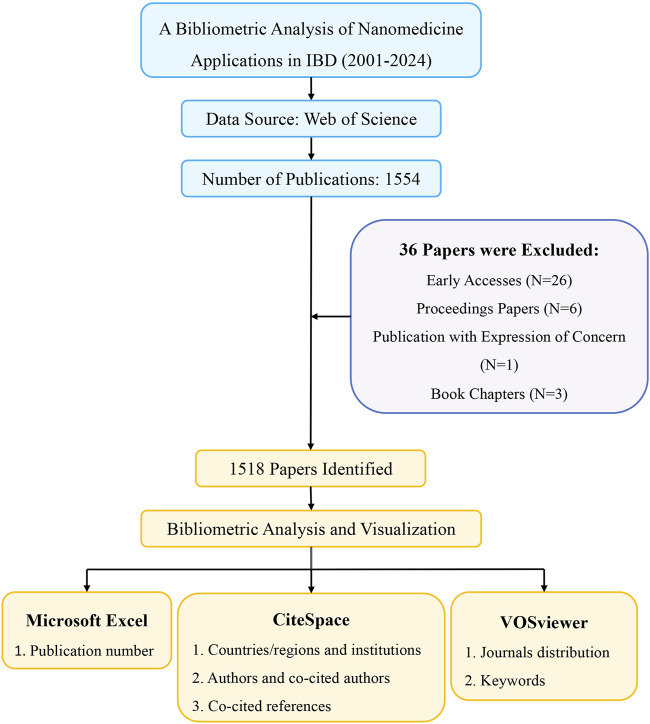
Flowchart for screening publications and conducting bibliometric analysis.

The downloaded files were imported into CiteSpace (version 6.3.R3), VOSviewer (version 1.6.18), and Microsoft Excel 2021 (version 16.48) for bibliometric visualization analysis. CiteSpace, developed by Prof. Chao-mei Chen, is a Java application for bibliometric analysis and visualization (Chen, 2004). In our study, CiteSpace was applied to analyze and visualize national and institutional contributions, authors and co-cited authors, co-cited reference clusters and timelines, keywords, and keywords burst. The specific settings of CiteSpace were established as follows: time slicing was from January 2001 to July 2024, with each slice representing 1 year. The term sources and links were set to the default parameters. VOSviewer is another bibliometric analysis software developed by Nees Jan van Eck and Ludo Waltman, which constructs visual network maps based on data (van Eck and Waltman, 2010). We used VOSviewer software to generate core authors, countries, research institutions, and keyword co-occurrence networks. For keywords and co-cited journals, the minimum occurrence frequency was set at 100 and 5, respectively. In addition, visualization of the annual publication numbers was performed using Microsoft Excel 2021. The procedure for data collection and bibliometric analysis are shown in Figure 1.

## 3 Results

### 3.1 Quantitative analysis of annual publication output

**FIGURE 2:**
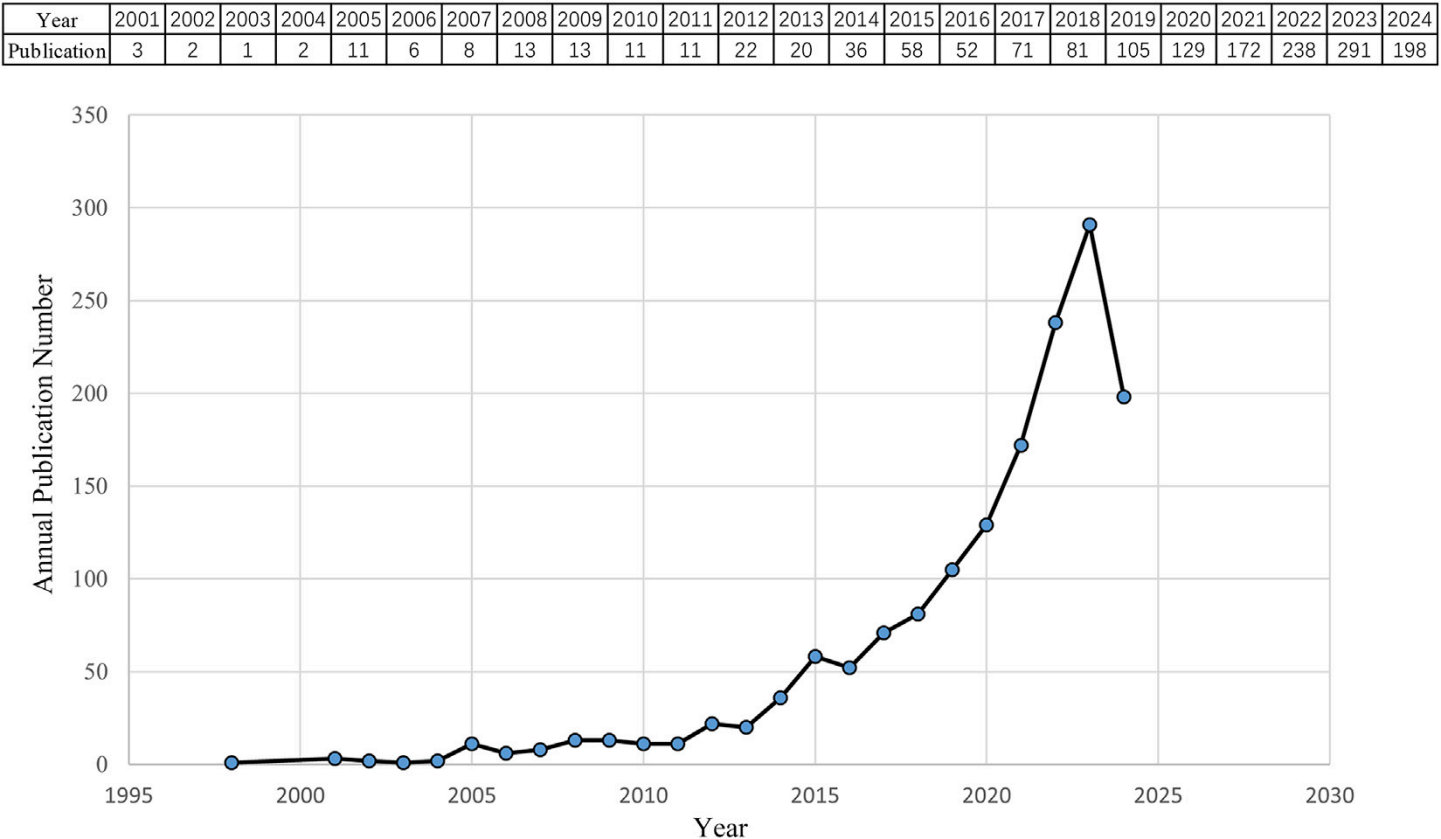
Quantity and trend in publication output for nanomedicine applications in IBD from 2001 to 2024.

According to the search criteria, a total of 1,554 pieces of literature were identified from the WoSCC database. 36 irrelevant records were screened out based on the exclusion criteria of document type and language, including 26 early accesses, 6 proceedings papers, 1 publication with expression of concern, and 3 book chapters. Ultimately, 1,518 studies, comprising 1,248 articles and 270 reviews, were collected for the final analysis. Figure 2 shows the annual number of papers published related to nanomedicine applications in IBD from 2001 to 2024. With the exception of 11 articles published in 2005, the annual academic output remained below 10 in the initial phase lasting from 2001 to 2007. During the subsequent phase, the output of publications overall continuously increased in a fluctuating manner. Strikingly, the number of papers rose rapidly from 2019 to 2023, peaking at 291 scholarly articles published in 2023. Based on these results, we anticipate that the number of annual publications will persistently increase, indicating that studies on nanomedicine applications in IBD are gaining interest.

### 3.2 Distribution of countries/regions and institutions

**FIGURE 3:**
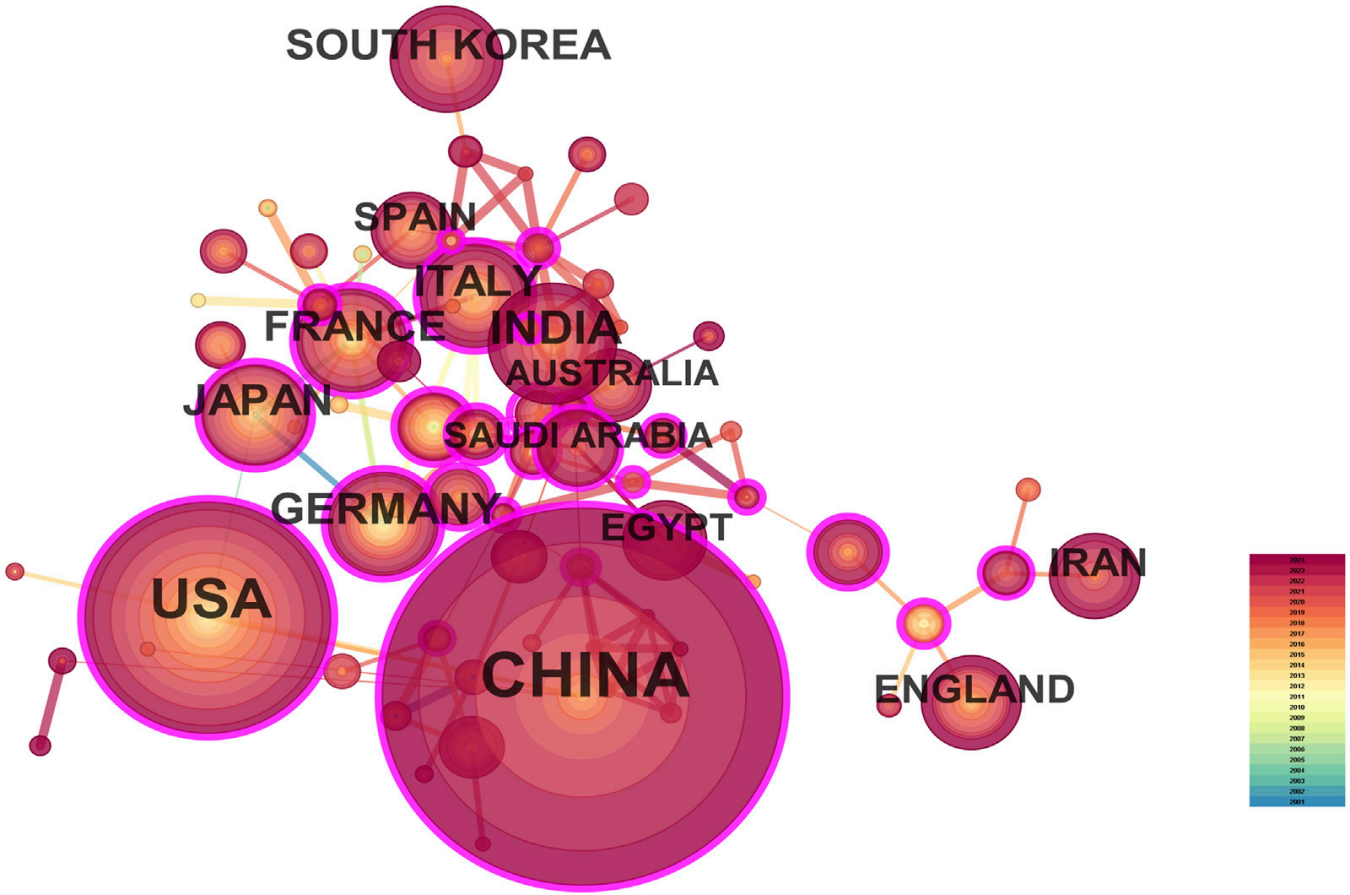
The visualization map of leading countries contributing to research on nanomedicine applications in inflammatory bowel disease (IBD).

**FIGURE 4:**
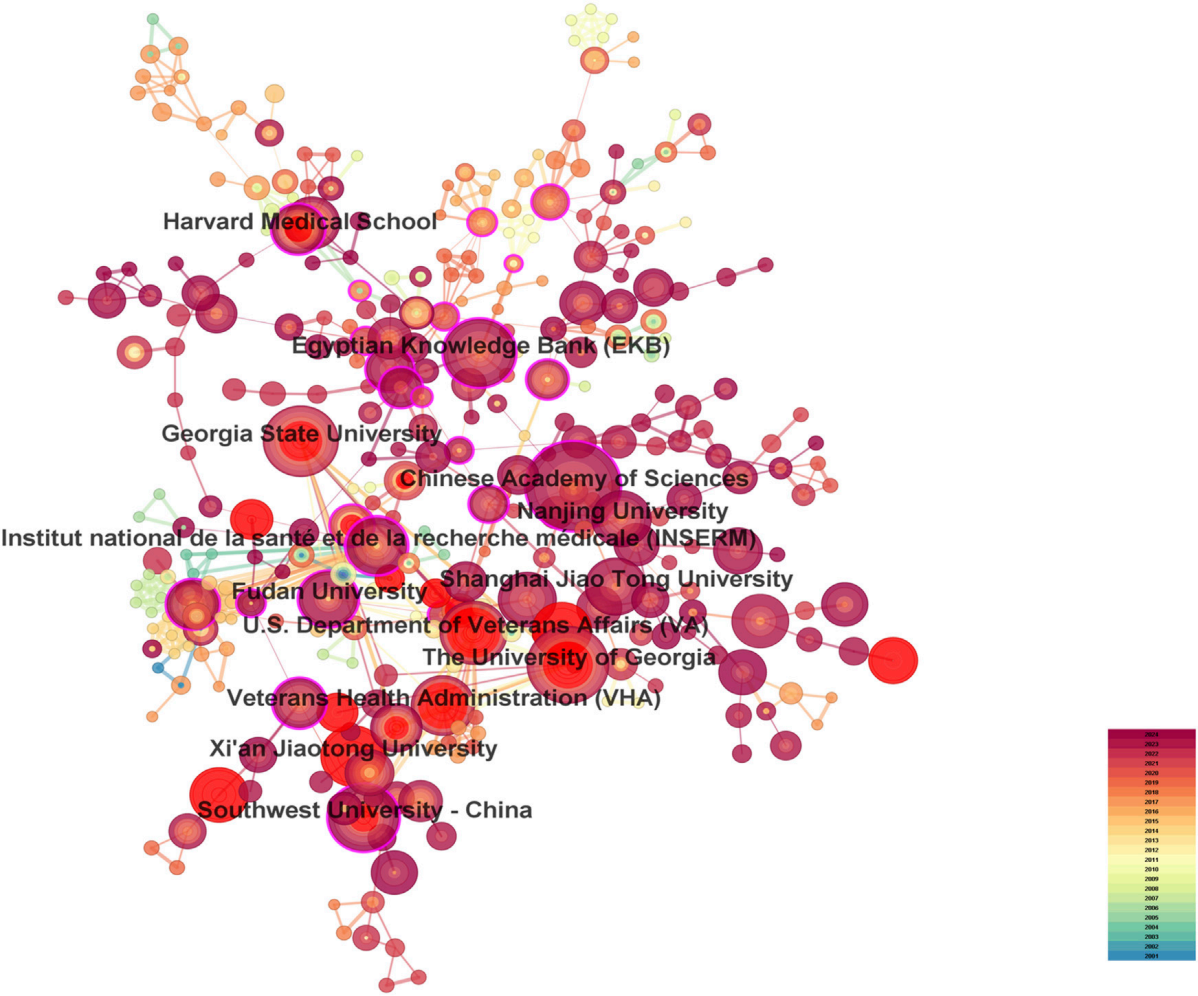
The visualization map of leading institutions contributing to research on nanomedicine applications in IBD.

A total of 287 countries/regions and 5,459 institutions have contributed to the field of IBD nanomedicine. The top 10 most productive countries/regions and institutions are summarized in Table 1. The results show that Germany (2001), Japan (2001), Italy (2001), France (2001), the United States (2002) started research in this field earlier, establishing a strong foundation for future advancements. Although China (2007) started relatively late, its development has been rapid, contributing the largest volume of publications (712, 45.8%), significantly higher than other countries/regions, followed by the United States (305, 19.6%), and India (107, 6.9%). The output from other countries/regions in the top 10 list have fewer than 90 publications each. Findings from Figure 3 reveal that China is a leading cooperation center (centrality = 0.33) in this field, with the United States (0.25), Italy (0.16), and France (0.15) ranking second, third, and fourth respectively. The top 10 most productive institutions are distributed across 4 countries, with 4 located in China and 4 in the United States. The three institutions that publishing the most relevant papers are the Chinese Academy of Sciences (70, 4.5%), the University of Georgia (55, 3.5%), and Georgia State University (51, 3.3%). Although the Egyptian Knowledge Bank (EKB) from Egypt ranks fourth (44, 2.8%), it has the highest centrality (0.22), closely followed by the Chinese Academy of Sciences (0.20), indicating that these institutions occupy significant positions in the research of the IBD nanomedicine (Figure 4).

**TABLE 1:** Publications in the 10 most productive countries/regions and institutions.

Rank	Country/Regions	Year	Count (%)	Centrality	Institutions	Year	Count (%)	Centrality
1	China	2007	712 (45.8)	0.33	Chinese Academy of Sciences	2017	70 (4.5)	0.2
2	United States	2002	305 (19.6)	0.25	The University of Georgia	2010	55 (3.5)	0.01
3	India	2005	107 (6.9)	0.09	Georgia State University	2012	51 (3.3)	0.01
4	Germany	2001	84 (5.4)	0.1	Egyptian Knowledge Bank (EKB)	2013	44 (2.8)	0.22
5	Japan	2001	81 (5.2)	0.05	Southwest University - China	2015	43 (2.8)	0.02
6	Italy	2001	77 (5.0)	0.16	U.S. Department of Veterans Affairs (VA)	2010	41 (2.6)	0.04
7	France	2001	75 (4.8)	0.15	Veterans Health Administration (VHA)	2012	38 (2.4)	0.03
8	South Korea	2013	73 (4.7)	0.05	Xi’an Jiaotong University	2020	33 (2.1)	0.02
9	England	2006	51 (3.3)	0.11	Shanghai Jiao Tong University	2018	32 (2.0)	0.03
10	Iran	2011	50 (3.2)	0.03	Institut national de la santé et de la recherche médicale (INSERM)	2004	29 (1.9)	0.13

### 3.3 Analysis of authors and co-cited authors

**FIGURE 5:**
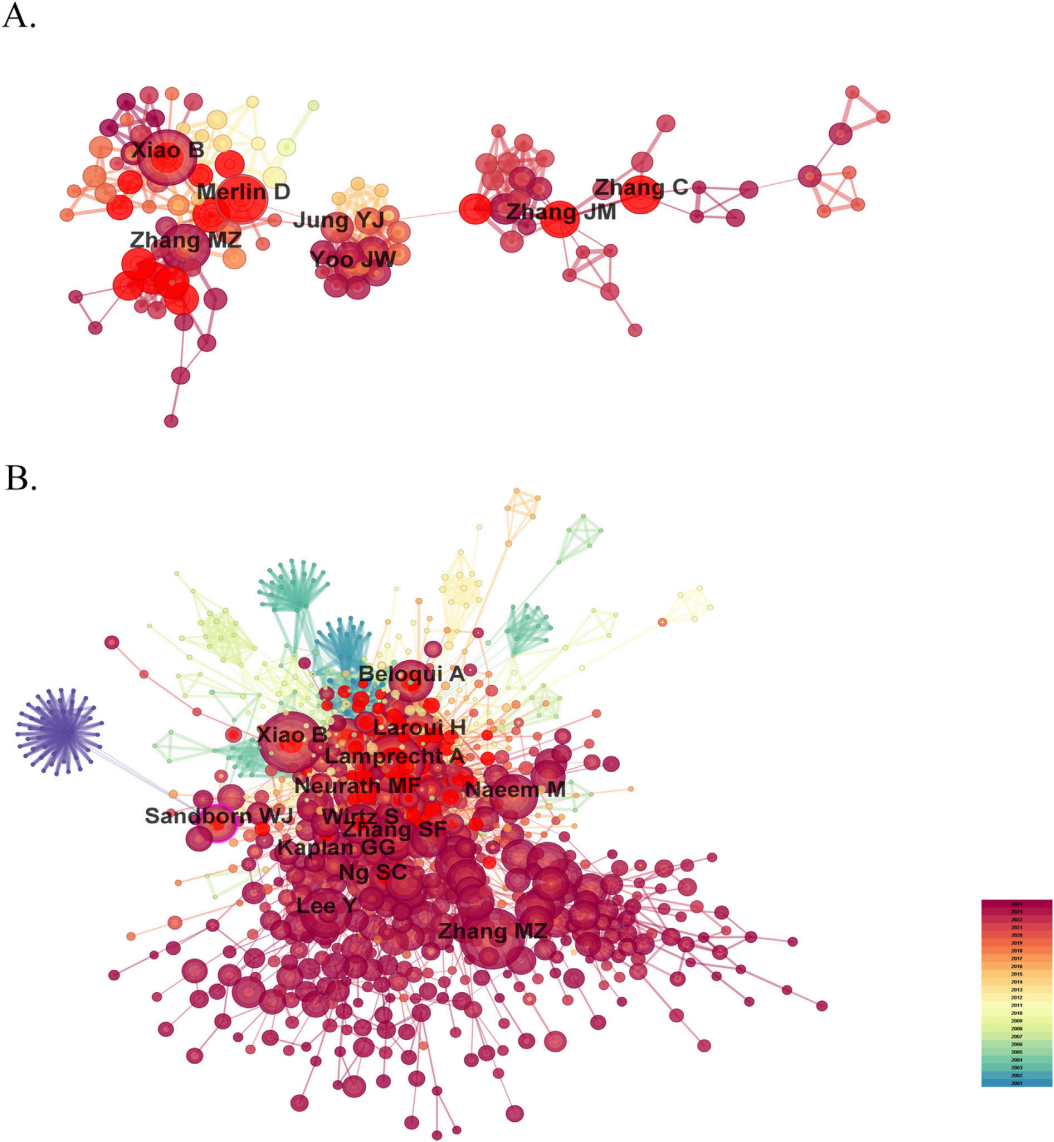
The visualization map of influential authors **(A)** and co-cited authors **(B)** in the field of nanomedicine applications in IBD.

Research on nanomedicine applications in IBD has seen contributions from 9,334 scholars, and the top 10 productive authors and co-cited authors are list in Table 2. Among these top authors with the most published papers, half are from China, two are from the United States, two from South Korea, and one from Germany. In terms of high productivity, Bo Xiao (Southwest University, China) leads with 37 literature, right after whom Didier Merlin (Georgia State University, United States) with 32, and Mingzhen Zhang (Georgia State University, United States) with 28. The other authors in the top 10 list have fewer than 20 documents each (Figure 5A). We cannot ignore that the centralities of all authors are less than 0.01, indicating that these researchers should strengthen cooperation with others. Co-cited author analysis examines instances where the works of two authors are simultaneously cited by a third author. A greater number of co-citations suggests that the authors have similar academic interests and that their research is closely aligned (Cheng et al., 2021). The top 10 co-cited authors were referenced more than 100 times each. The most frequency co-cited author is Bo Xiao, with 260 citations, who is also the most productive author (Figure 5B). Following him are Alf Lamprecht (246 citations; University of Bonn, Germany), Mingzhen Zhang (232 citations), and Hamed Laroui (168 citations; Georgia State University, United States). China has not only produced the largest number of publications but also has the most productive and frequently co-cited authors, highlighting its rapid advancement in this research area despite a relatively late start.

**TABLE 2:** Top 10 authors and co-cited authors.

Rank	Author	Count	Centrality	Co-cited author	Citations	Centrality
1	Xiao B	37	0.01	Xiao B	260	0.1
2	Merlin D	32	0.01	Lamprecht A	246	0.12
3	Zhang MZ	28	0	Zhang MZ	232	0.08
4	Lamprecht A	19	0	Laroui H	168	0.06
5	Zhang C	15	0.01	Ng SC	152	0.01
6	Zhang JM	15	0.01	Zhang SF	151	0.03
7	Yoo JW	14	0	Lee Y	150	0.01
8	Jung YJ	13	0.01	Neurath MF	147	0.04
9	Yang CH	12	0.01	Kaplan GG	138	0.01
10	Yang M	11	0	Naeem M	131	0.02

### 3.4 Analysis of journals distribution

**FIGURE 6:**
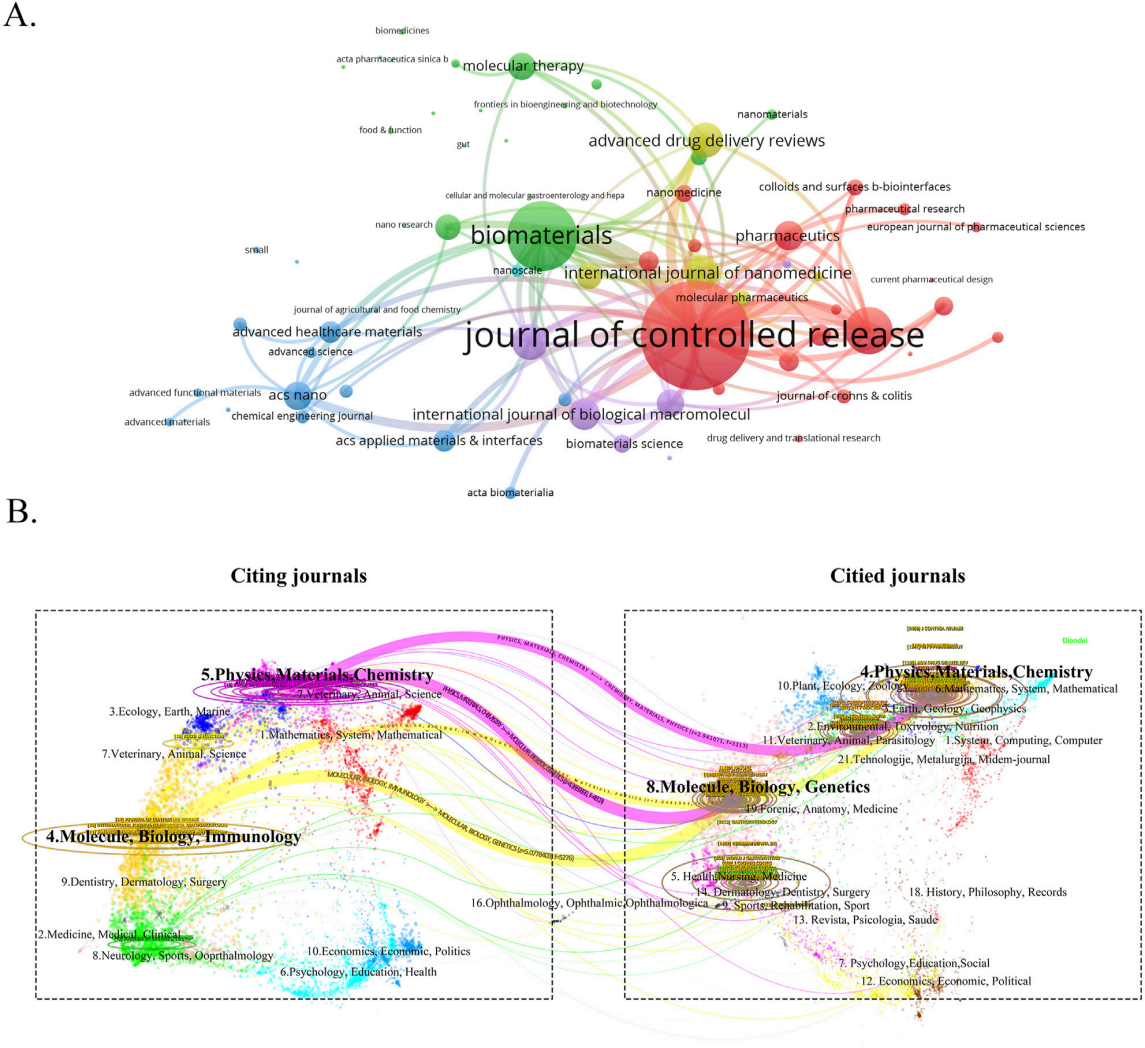
**(A)** The visualization map of journals publishing papers on nanomedicine applications in IBD. **(B)** The dual-map overlay of the relevant journals.

To gain a comprehensive understanding of the publication landscape and citation dynamics within the field of nanomedicine applications for IBD, we investigated the distribution of documents across various journals and disciplines. All papers on nanomedicine applications in IBD are sourced from 447 journals. As shown in Table 3, the *Journal of Controlled Release* (IF 2023 = 10.5), a renowned journal in pharmacy, published the most papers, followed by *International Journal of Pharmaceutics* (IF 2023 = 5.8), and *International Journal of Biological Macromolecules* (IF 2023 = 7.7) (Figure 6A). In order to determine the causal relationships between journal citations, we used a dual-map overlay of relevant journals to visualize the citation paths across related fields. Based on Figure 6B, we note that the analyzed papers are predominantly distributed across the fields of Physics, Materials Chemistry, and Molecule, Biology, Immunology, with their cited papers also primarily found in the similar disciplines. Table 4 presents details on the top 10 co-cited journals in the field of nanomedicine applications in IBD. Among these, the United States and the Netherlands contribute four journals each, and the UK has two. Notably, the most co-cited journals are from medical-related fields, *Gastroenterology* (IF 2023 = 10.5, United States) and *Gut* (IF 2023 = 10.5, UK), the top two journals in gastroenterology, are ranked first (1,247 citations) and fourth (893 citations), respectively. In contrast, there is only one journal from the materials science field: *Biomaterials* (IF 2023 = 12.8, United States), which is ranked third with 903 citations.

**TABLE 3:** The top 10 journals with the most publications.

Rank	Journal	Count	Citation	Total link strength	JCR	IF (2023)
1	Journal of Controlled Release	52	2,549	1,207	Q1	10.500
2	International Journal of Pharmaceutics	43	1,391	527	Q1	5.800
3	International Journal of Biological Macromolecules	35	438	348	Q1	7.700
4	International Journal of Nanomedicine	34	829	390	Q1	6.600
5	Biomaterials	31	2,309	767	Q1	12.800
6	Pharmaceutics	31	545	329	Q1	4.900
7	ACS nano	27	714	319	Q1	15.800
8	Journal of Nanobiotechnology	27	486	287	Q1	10.600
9	Journal of Drug Delivery Science and Technology	27	277	312	Q1	4.500
10	Advanced Healthcare Materials	26	504	246	Q1	10.000

**TABLE 4:** Document types of the publications.

Rank	Category	Count	Co-cited journal	Citation	JCR	IF(2023)
1	Pharmacology & Pharmacy	433	Gastroenterology	1,247	Q1	25.700
2	Nanoscience & Nanotechnology	280	Journal of Controlled Release	1,035	Q1	10.500
3	Chemistry, Multidisciplinary	257	Biomaterials	903	Q1	12.800
4	Materials Science, Multidisciplinary	185	Gut	893	Q1	23.000
5	Materials Science, Biomaterials	180	Inflammatory Bowel Diseases	790	Q1	4.500
6	Biochemistry & Molecular Biology	161	International Journal of Pharmaceutics	768	Q1	5.800
7	Chemistry, Physical	106	Nature	755	Q1	50.500
8	Engineering, Biomedical	105	Plos One	745	Q1	2.900
9	Medicine, Research & Experimental	104	Advanced Drug Delivery Reviews	718	Q1	15.200
10	Gastroenterology & Hepatology	83	Proceedings of the National Academy of Sciences of the United States of America	714	Q1	9.400

### 3.5 Co-cited references analysis and clustering network

**FIGURE 7:**
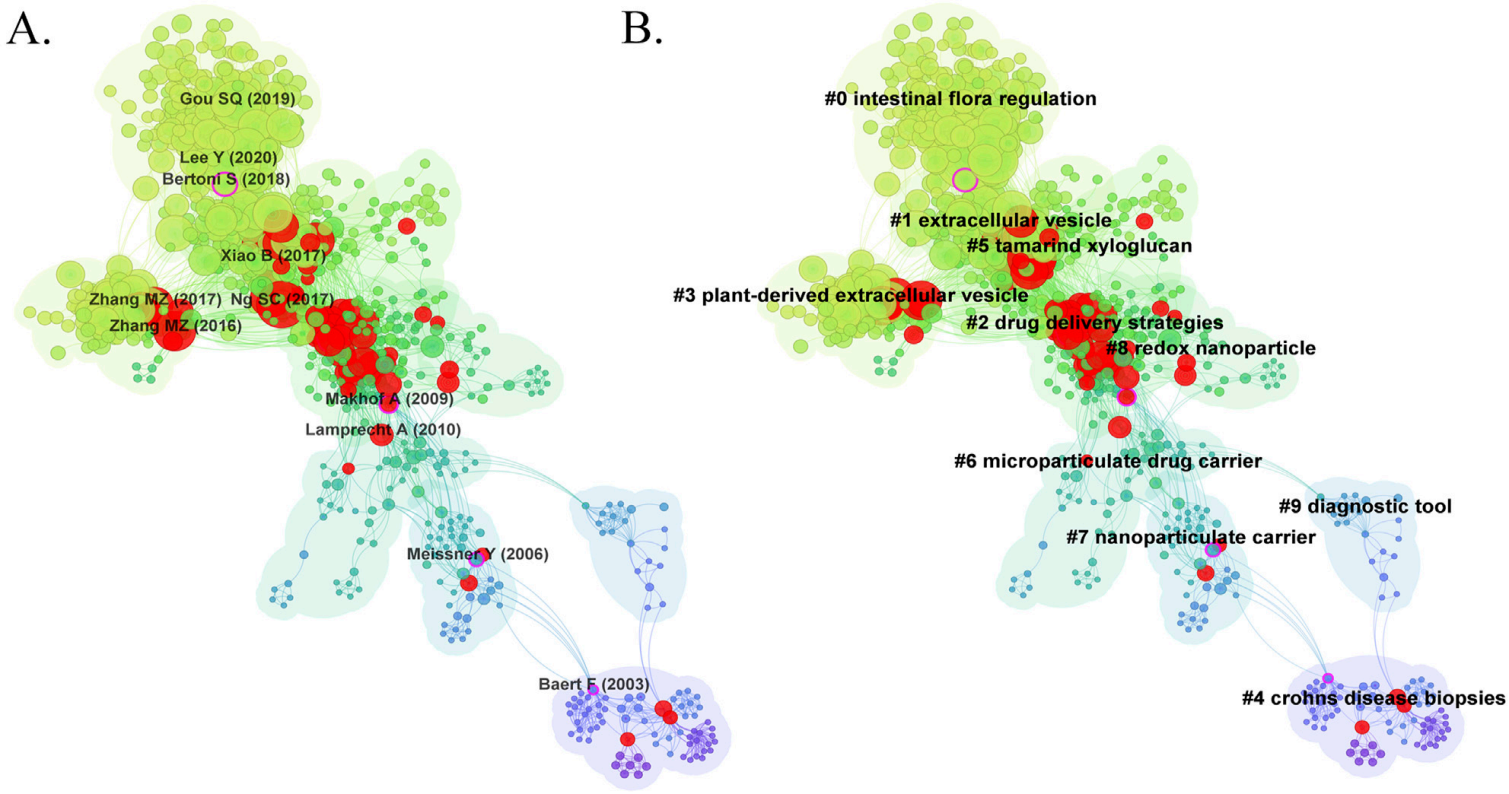
**(A)** The visualization map of co-cited references on nanomedicine applications in IBD. **(B)** The clustering network map of co-cited references.

Literature co-citation analysis reveals the relationship between documents by examining the frequency at which they are cited (Yang et al., 2020). A total of 1,119 co-cited references in the research of nanomedicine applications in IBD were visualized, and the first authors of the top 11 most co-cited references are depicted in Figure 7A. The size of the circles is proportional to the co-citation frequency, and the redder the color, the more co-citations it has within the time zone. Additionally, a complete list of the top 10 co-cited references is present in Table 5. Among these publications, only one study is a systematic review, ranked third, while the other 9 studies are clinical trials. All references were co-cited at least 50 times. The top 2 highly co-cited are as follows: The most co-cited article is ‘Hyaluronic Acid-bilirubin Nanomedicine for Targeted Modulation of Dysregulated Intestinal Barrier, Microbiome and Immune Responses in Colitis’ published in *Nature Materials* with 125 citations, authored by (Lee et al., 2020). The second article, published in *Biomaterials* by Gou et al. (2019), is titled “Multi-bioresponsive Silk Fibroin-based Nanoparticles with On-demand Cytoplasmic Drug Release Capacity for CD44-Targeted Alleviation of Ulcerative Colitis.” These two original articles introduce the biocompatibility and targeted delivery ability of nanomaterials, demonstrating that nanomedicine can improve the therapeutic efficiency of IBD by maintaining gut microbiome homeostasis and regulating the innate immune response. The results of these studies lay the foundation for further research on nanomedicine applications in IBD. The third co-cited reference is ‘Worldwide Incidence and Prevalence of Inflammatory Bowel Disease in the 21st Century: A Systematic Review of Population-based Studies’ published in *Lancet* by (Ng et al., 2017). This systematic review is widely cited because it offers comprehensive data on IBD incidence and prevalence from a global perspective, with significant implications for public health policy and clinical practice.

**TABLE 5:** Top 10 co-cited references.

Ranks	Title	Journal	Co-citation
1	Hyaluronic acid-bilirubin nanomedicine for targeted modulation of dysregulated intestinal barrier, microbiome and immune responses in colitis	Nature Materials	125
2	Multi-bioresponsive silk fibroin-based nanoparticles with on-demand cytoplasmic drug release capacity for CD44-targeted alleviation of ulcerative colitis	Biomaterials	69
3	Worldwide incidence and prevalence of inflammatory bowel disease in the 21st century: a systematic review of population-based studies	Lancet	69
4	Orally Targeted Delivery of Tripeptide KPV via Hyaluronic Acid-Functionalized Nanoparticles Efficiently Alleviates Ulcerative Colitis	Molecular Therapy	64
5	Plant-Derived Exosomal MicroRNAs Shape the Gut Microbiota	Cell Host & Microbe	53
6	A Proresolving Peptide Nanotherapy for Site-Specific Treatment of Inflammatory Bowel Disease by Regulating Proinflammatory Microenvironment and Gut Microbiota	Advanced Science	53
7	Oral Delivery of Nanoparticles Loaded with Ginger Active Compound, 6-Shogaol, Attenuates Ulcerative Colitis and Promotes Wound Healing in a Murine Model of Ulcerative Colitis	Journal Of Crohn’s and Colitis	53
8	An Orally Administered CeO_2_@Montmorillonite Nanozyme Targets Inflammation for Inflammatory Bowel Disease Therapy	Advanced Functional Materials	52
9	Edible ginger-derived nanoparticles: A novel therapeutic approach for the prevention and treatment of inflammatory bowel disease and colitis-associated cancer	Biomaterials	52
10	Broccoli-Derived Nanoparticle Inhibits Mouse Colitis by Activating Dendritic Cell AMP-Activated Protein Kinase	Molecular Therapy	51

**FIGURE 8:**
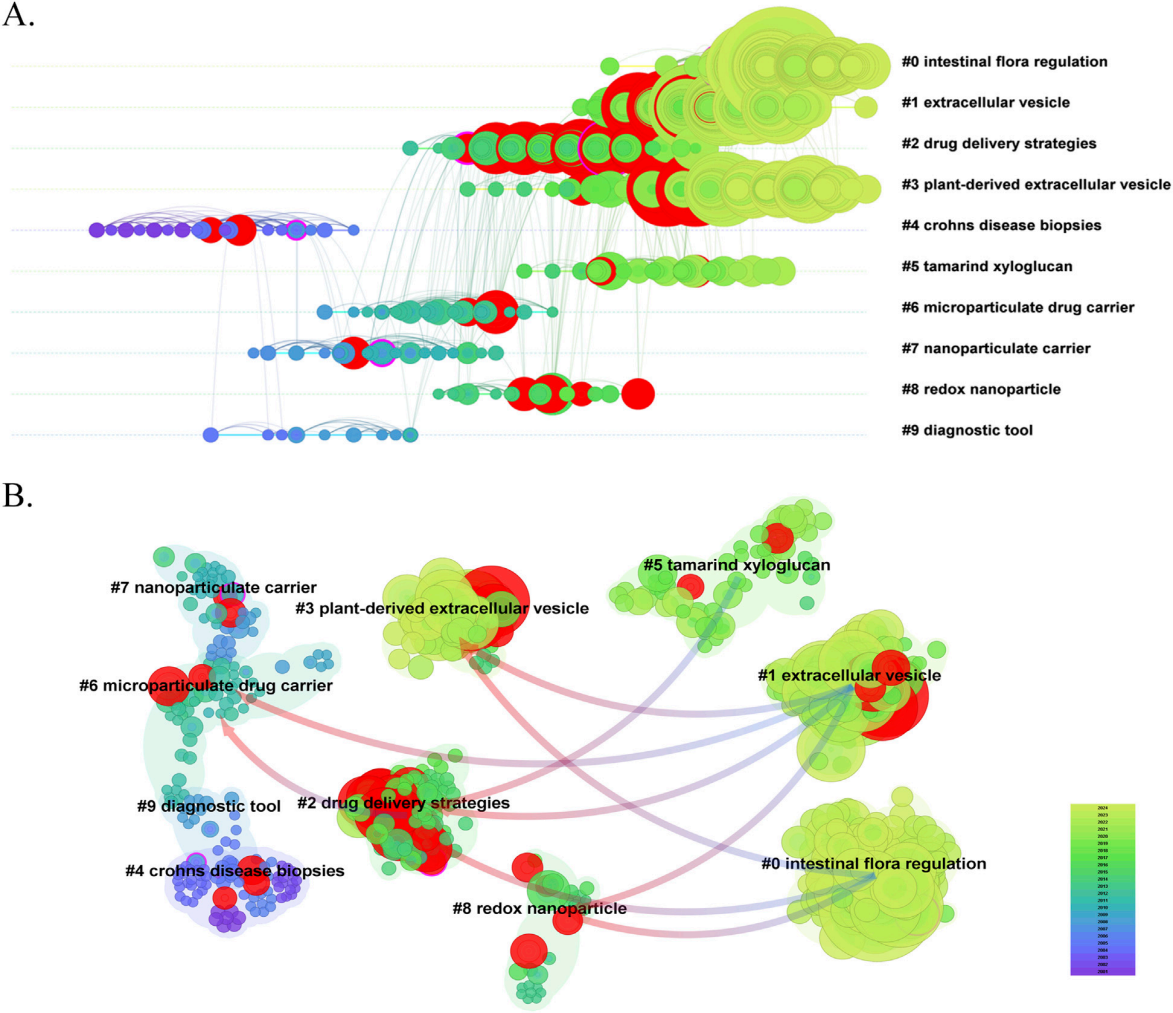
**(A)** The timeline view of co-citation clusters. **(B)** The dependency relationships among different co-citation clusters.

Cluster analysis of literature co-citation objectively reveals the knowledge structure of the research field (Yang et al., 2019). We further generated a visual map of the top 10 clusters based on the co-cited references (Figure 7B). Cluster #0, identified as intestinal flora regulation, was the largest cluster, followed by extracellular vesicle (cluster #1), drug delivery strategies (cluster #2), plant-derived extracellular vesicle (cluster #3), Crohn’s disease biopsies (cluster #4), tamarind xyloglucan (cluster #5), microparticulate drug carrier (cluster #6), nanoparticulate carrier (cluster #7), redox nanoparticle (cluster #8), and diagnostic tool (cluster #9). As shown in Figure 8A, we conducted a timeline of co-cited references to visualize the evolution of research trends and hotspots over time. As we have seen, cluster #4 and cluster #9 began study earlier, cluster #2 has a high concentration of nodes with citation bursts in recent years, and cluster #0, #1, #3, and #5 are considered research fronts as they are still ongoing. Figure 8B illustrates the dependency relationships between different research clusters, highlighting the mutual influence and citation relationships among them. Consistent with Figure 8A, cluster #2 serves as a significant reference basis for subsequent research, while cluster #0, #1, and #5 represent the current frontiers of research. This graph indicates that in the study of intestinal flora regulation, research on cluster #2, #3, and #8 serve as important reference foundation; in the study of extracellular vesicles, research on cluster #2, #3, #6, and #8 provide crucial reference foundation; similarly, in the study of tamarind xyloglucan, research on cluster #2 serves as an important reference foundation.

### 3.6 Keywords co-occurrence analysis of research hotspots

**FIGURE 9:**
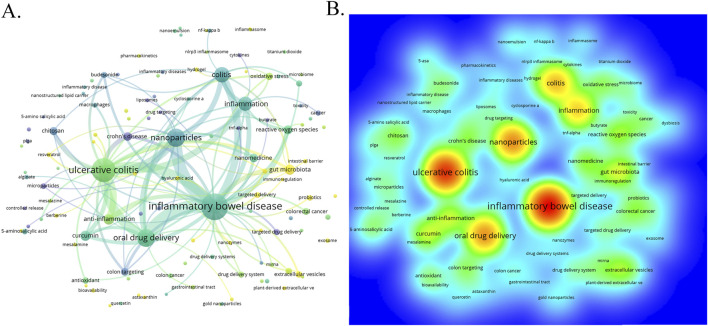
**(A)** The visualization map of keywords co-occurrence network on nanomedicine applications in IBD. **(B)** The heatmap of keywords.

**FIGURE 10:**
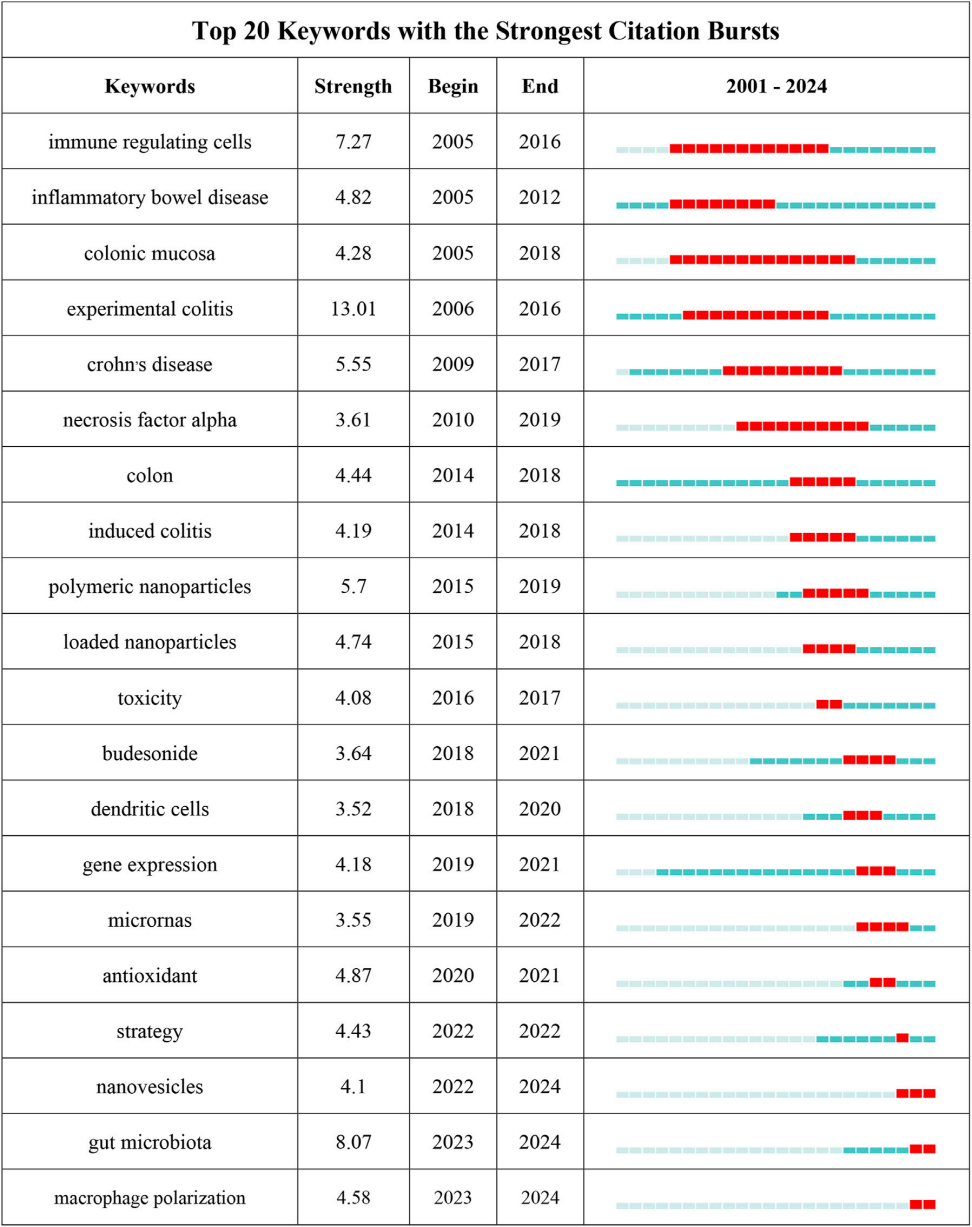
The top 25 keywords with the strongest citation bursts on nanomedicine applications in IBD.

Through the co-occurrence analysis of keywords (Figures 9A, B), we can quickly capture the current hotspots and future research directions in this discipline (Wu et al., 2022). A total of 3,433 keywords were extracted from these papers, with 52 keywords appearing more than 10 times. Table 6 presents the top 20 high-frequency keywords in research on nanomedicine applications in IBD. Among these keywords, “inflammatory bowel disease” (n = 367) and “ulcerative colitis” (n = 294) are the most frequently used terms, followed by “nanoparticles” with 172 occurrences. Additionally, the total link strengths, which refers to the total number of co-occurrences of a keyword with others (Cai et al., 2023), for these three keywords exceed 160. We analyzed the top 20 keywords with the strongest citation bursts from 2001 onwards (Figure 10). As indicated by the red segments, these keywords experienced a blowout in usage at this period. The keyword “immune regulating cells” has the strongest burst (burst strength 7.27). The keywords “colonic mucosa” (burst duration from 2005 to 2018, 13 years) and “immune regulating cells” (2005–2016, 11 years) receive the most sustained attention over time. Furthermore, we also found that “nanovesicles,” “gut microbiota,” and “macrophage polarization” have emerged more recently, revealing that these topics might represent current hotspots in the field of nanomedicine applications in IBD.

**TABLE 6:** The top 20 keywords.

Rank	Keyword	Occurrences	Total link strength	Rank	Keyword	Occurrences	Total link strength
1	inflammatory bowel disease	367	311	11	nanomedicine	39	37
2	ulcerative colitis	294	240	12	crohn’s disease	37	35
3	nanoparticles	172	162	13	chitosan	35	34
4	oral drug delivery	163	143	14	colon targeting	31	27
5	colitis	124	109	15	oxidative stress	29	25
6	inflammation	108	98	16	colorectal cancer	25	19
7	anti-inflammation	58	53	17	extracellular vesicles	24	22
8	gut microbiota	56	50	18	antioxidant	22	20
9	curcumin	55	49	19	exosomes	22	19
10	reactive oxygen species	42	39	20	budesonide	21	21

## 4 Discussion

In this era of information overload, staying on the top of the industry developments and keeping informed about the latest research findings is increasingly challenging (Liu L. et al., 2022). To present the current global intellectual base and research trends of nanomedicine applications in IBD, we gathered scholarly articles from 2001 to 2024 and conducted a bibliometric analysis to visualize the knowledge structures within this specific field.

### 4.1 General information

As of 15 July 2024, a total of 1,518 publications are included, with contributions from 9,334 authors across 447 journals, 5,459 institutions and 287 countries/regions. Alf Lamprecht is considered a pioneer in nanomedicine applications for IBD. His seminal 2001 paper investigated the size-dependent deposition of microparticles and nanoparticles in a rat model of experimental colitis, establishing the foundation that nano-sized carriers could enhance targeted drug delivery to inflamed regions in IBD (Lamprecht et al., 2001a). From 2001 to 2004, research on nanomedicine applications in IBD was in its infancy stage, with less than 3 publications per year. The field experienced a slow growth phase from 2005 to 2011, averaging 10.4 papers annually. However, from 2011 to 2024, the field entered a rapid growth phase, with a particularly notable surge in the past 6 years, where the number of annual publications consistently exceeded 100 each year. This trend suggests that research on nanomedicine applications in IBD is currently in an explosive period and is likely to remain active in the coming years as targeted medicine continue to gain prominence among scholars (Takedatsu et al., 2015).

China and the United States are the leading countries in research on nanomedicine applications in IBD, contributing the greatest output of publications. The United States began research in this field earlier, establishing a leading position and laying a strong foundation for future advancements. In contrast, China entered the field later but has rapidly become a dominant contributor, gradually solidifying its position. China not only produces the highest number of publications but has also emerged as a central hub for international collaborations in recent years, with a centrality score of 0.33. With the exception of one institution from Egypt and one from France, the remaining 8 institutions in the top 10 most productive are all located in China and the United States. The Global Burden of Disease Study 2019 revealed that China and the United States had the highest number of prevalent cases (Wang et al., 2023). Therefore, the incidence of IBD is a contributing factor to their dominance in IBD-related publications, particularly in the United States, where the disease burden is high, and in China, where the incidence has been rapidly rising in recent years. Additionally, the distribution of institutions and collaboration network explain why these two nations involved in the most papers in this field. Their strong academic infrastructures, substantial government funding, and international collaborations contributed to the growth of IBD research and implies that establishing premier research institutions is crucial for elevating the prominence of the academic community (Pei et al., 2022).

Research on nanomedicine applications in IBD was spearheaded by Alf Lamprecht, an influential expert in drug development. Dr. Lamprecht initially focused on the preparation and structural analysis of microparticles and nanoparticles. In 2001, he published 2 trailblazing experimental studies on the applications of nanoparticles in IBD treatment (Lamprecht et al., 2001a; Lamprecht et al., 2001b). His work introduced the potential of nanoparticles for targeted drug delivery to inflamed colonic mucosal areas, demonstrating that this approach is more effective than traditional drug solution, with the added benefit of minimizing systemic side effects and preventing relapse. Alf Lamprecht laid the foundation for nanomedicine applications in IBD. In subsequent studies, Dr. Lamprecht enhanced the therapeutic efficacy of nanocarriers encapsulating the drugs for IBD by incorporating PH-sensitive delivery mechanisms (Meissner et al., 2006) and implementing specific surface modifications (Wachsmann et al., 2013). Bo Xiao has not only published the greatest number of related papers but also ranks as the top co-cited authors, highlighting his prominent contribution to this field. The research of Dr. Xiao primary focused on developing advanced drug delivery systems for IBD. The initial work involved creating foundational nanoparticle systems, including a mannosylated bioreducible cationic polymer that designed to efficiently targets macrophages and inhibit TNF-α expression, thereby effectively treating IBD (Xiao et al., 2013). As the research advanced, the team incorporated more sophisticated materials and methods, such as PH-sensitive materials (Xiao et al., 2015) and functional modifications (Xiao et al., 2014b), to optimize nanoparticle performance, improving the accumulation and action of drugs within specific cell types via enhancing drug release characteristics and cellular uptake efficiency. Dr. Xiao revealed that orally administered nanoparticles, functionalized with surface antibodies against CD98 and carrying CD98 siRNA, could significantly reduce CD98 expression in colonic epithelial cells and macrophage, thereby alleviating colitis severity in mice (Xiao et al., 2014a). They also demonstrated that Hyaluronic Acid-Functionalized nanoparticles (HA-NPs) loaded with Lysine-proline-valine could effectively target colonic cells, promote mucosal healing and reduce inflammation in IBD (Xiao et al., 2017). As their research progressed, they attention gradually shifted toward combination therapies, such as using HA-NPs to co-deliver CD98 siRNA and the anti-inflammatory agent curcumin, thus enhancing therapeutic efficacy through the combined strategy of multiple drugs (Xiao et al., 2016). At the same time, Bo Xiao has cooperated with Didier Merlin (ranks 2ed among the top 10 authors), Mingzhen Zhang (3rd), Jinming Zhang (6th), and Yunjin Jung (8th), resulting in several high-level publications, demonstrating that the development of a team relies heavily on collaborative efforts.

The analysis of journal sources for documents on nanomedicine applications in IBD reveals a distribution across various academic fields. Most of these publications are concentrated in journals within pharmacy, chemistry, and materials science, while medical-related fields ranking lower, occupying the ninth and tenth positions. This indicates that the advancement of nanomedicine for IBD is predominantly driven by progresses in fundamental sciences, particularly in the design, synthesis, and applications of novel materials (Kong et al., 2023; Shahcheraghi et al., 2022). Interestingly, although the majority of these studies are published in materials and pharmaceutical science journals, the most frequently co-cited journals are from the medical fields, particularly those specializing in gastroenterology, such as *Gastroenterology* and *Gut*. This indicates the clinical significance of these foundational studies and suggests that while the research originates in material and chemical sciences, its most profound applications and impact are in the medical field (Fu et al., 2023; Yang and Merlin, 2019). Overall, these findings emphasize the interdisciplinary nature of this research area and underscore the critical need for continued collaboration between fundamental sciences and clinical medicine to advance the nanomedicine applications in IBD.

The cluster analysis of literature co-citation provides an insightful perspective on the evolving knowledge structure in the field of nanomedicine applications for IBD (Yuan et al., 2024). Cluster #4 (Crohn’s disease biopsies) and Cluster #9 (diagnostic tools) represent earlier research, highlighting foundational work in nanomedicine at the diagnostic level. In contrast, Cluster #2 (drug delivery strategies) has experienced a recent surge in citations, suggesting an increased emphasis on the development and refinement of therapeutic approaches in recent years. The largest cluster, Cluster #0 (intestinal flora regulation), and the most recent cluster Clusters #1 (extracellular vesicle), signify significant areas of focus within the field. Notably, Clusters #3 (plant-derived extracellular vesicle) has emerged as a new and unique research cluster, reflecting their growing recognition due to their distinctive bioactive properties, biocompatibility, and low immunogenicity (Rome, 2019; Mu et al., 2023). Plant-derived extracellular vesicles have become a promising and rapidly developing area of research in the treatment of IBD. Current research trends are increasingly concentrated on innovative drug delivery systems, extracellular vesicles, and the role of gut microbiota.

Co-cited references are regarded as the foundational research within a particular field (Pei et al., 2022). In this bibliometric study, we identified the 10 most co-cited references in the field of nanomedicine applications in IBD. Except for one review that assessed the changing incidence and prevalence of IBD globally in the 21st century (Ng et al., 2017), the remaining papers, all experimental studies, focus on the development and applications of natural and synthetic nanomedicines and their potential for widespread use in IBD treatment. There are 3 studies explore the potential of plant-derived nanoparticles in treating IBD by targeting specific pathways and mechanisms within the gut (Zhang et al., 2016; Deng et al., 2017; Teng et al., 2018). This novel and natural delivery mechanism open new therapeutic avenues for addressing IBD while overcoming the potential toxicity in traditional nanoparticles. Synthetic nanomedicines, with their multifunctional properties, not only can precisely target inflamed colon tissues but also restore gut barrier integrity through various mechanisms. For instance, hyaluronic acid-bilirubin nanomedicine (HABN) (Lee et al., 2020) and Multi-bioresponsive silk fibroin-loaded nanoparticles (Gou et al., 2019) have shown enhanced therapeutic efficacy by promoting gut microbiome homeostasis and modulating immune responses, making them promising candidates for IBD treatment. Li et al. (2019) developed a smart nanotherapy using oxidation-responsive nanoparticles that release a package proresolving peptide in response to elevated reactive oxygen species (ROS) at diseased sies. This approach effectively reduces inflammation and promotes gut healing while demonstrating safety in a mouse model, paving the way for further development of targeted precision therapies for IBD and other inflammatory diseases. Overall, the co-cited references primarily focus on drug delivery strategies, which are the research basis for nanomedicine treatment in IBD.

### 4.2 Hotspots and emerging frontiers

**FIGURE 11:**
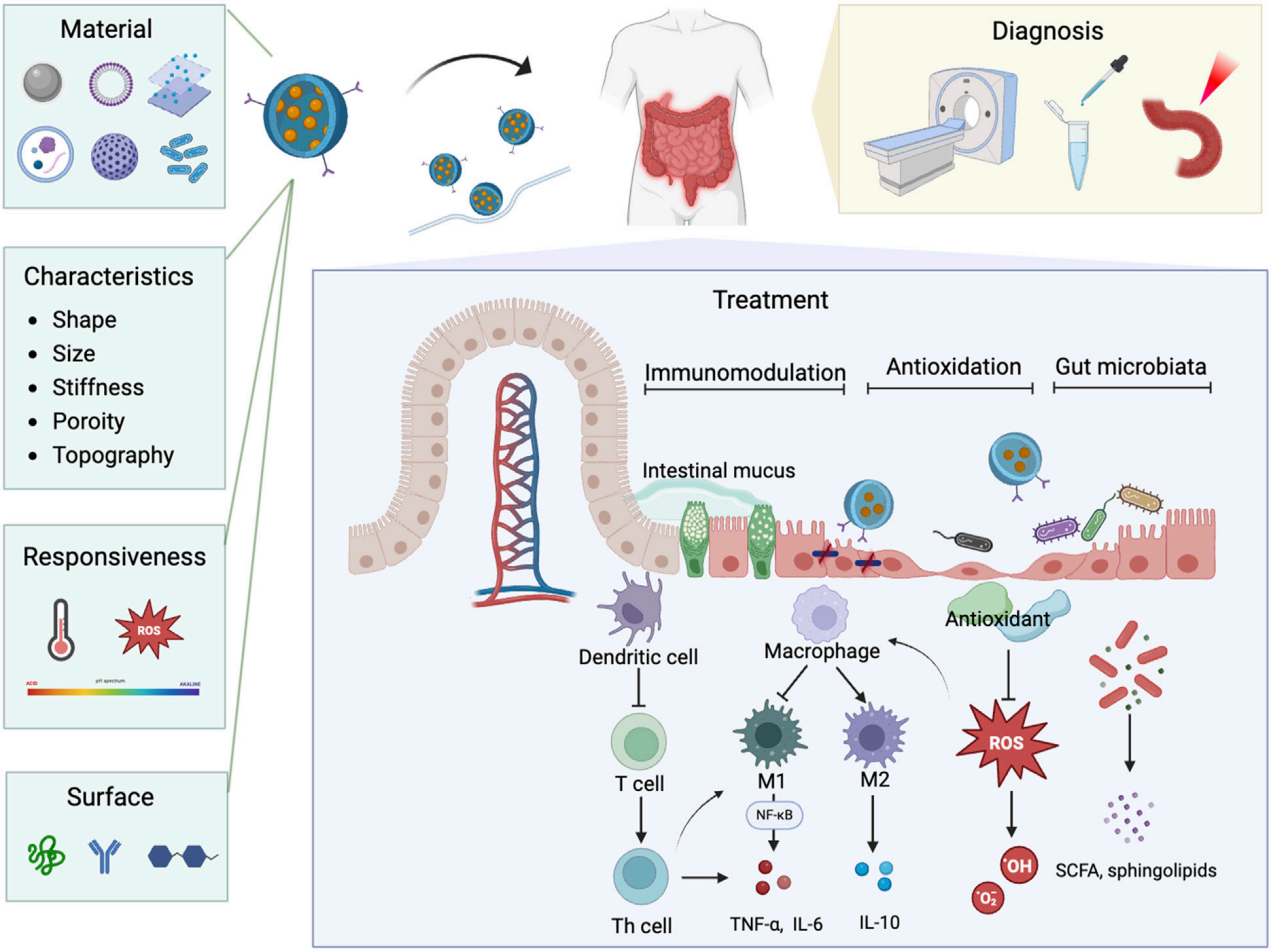
The schematic illustration of nanoparticle properties and their diverse applications in nanomedicine for IBD.

In bibliometrics, the occurrence and burst of keywords can reflect the emerging hotspots in a specific field, providing crucial insights into its development and growth (Liu et al., 2014). Excluding keywords such as IBD, colitis, and nanoparticles, the mainly keywords include drug delivery, immune regulating cells, antioxidants, gut microbiota, and nanovesicles. According to keywords clustering analysis and the strongest citation bursts, the hotspots and current frontiers in the field of nanomedicine applications in IBD can be summarized as follows (Figure 11):

Nanodelivery systems have transformed modern medicine by offering precise and efficient drug delivery to targeted tissues and cells. These systems can encapsulate therapeutic agents like probiotic (Xu C. et al., 2022), protecting them from degradation. Nanoparticles can be engineered to respond to specific stimuli, such as pH changes (Zhang et al., 2017), allowing for the controlled and sustained release of drugs directly at the colonic inflamed areas. Additionally, the surface of nanoparticles can be modified with specific ligands that recognize and bind to receptors on target cells, ensuring precise drug release at the desired locations (Lv et al., 2023). The targeting capabilities of nanotechnology also show great promise in the diagnosis of IBD. Nanoparticles can be engineered to specifically bind to inflamed tissues, allowing for more precise imaging and detection (Yue et al., 2023; Truffi et al., 2017). Integration with imaging modalities such as CT and MRI have enhanced early detection, disease activity monitoring, and assessment of therapeutic response (Expert Panel on Gastrointestinal et al., 2020; Yin et al., 2023). Multifunctional bio-nano platforms, including contrast agents, near-infrared fluorescent probes, and bioactive substance detection agents, have been developed to facilitate more accurate diagnosis and ongoing monitoring of IBD (Fu et al., 2023; Zhou D. et al., 2022; Assadsangabi et al., 2024). This precision in targeting not only improves diagnostic accuracy but also aids in monitoring disease progression and evaluating the effectiveness of treatments at a molecular level. Encouragingly, high specific nanoparticles designed to target molecular and cellular processes associated with IBD can simultaneously treat the disease while tracking its progression (Yin et al., 2023; Yan et al., 2022; Elinav and Peer, 2013). This targeted delivery positions nanodelivery systems as promising tool for advanced medical diagnostics and therapies for IBD.

Nanodelivery systems, with their precision targeting and controlled release capabilities, not only enhance drug delivery but also lay a crucial foundation for advancing immunotherapy approaches in IBD treatment. Nanotechnology can target specific inflamed tissues and immune cells with precision, effectively modulating immune system. One of the key advantages of nanoparticles in IBD therapy is their ability to specifically target immune-regulating cells, which are overactive in IBD and lead to chronic inflammation and tissue damage (Hou et al., 2020; Saez et al., 2023). CD44-targeted nanoparticles can actively target inflammatory colonic epithelial cells and macrophages (Lv et al., 2023; Vafaei et al., 2016), improving the uptake of encapsulated drugs, thereby suppressing macrophage proliferation and downregulating the secretion of pro-inflammatory cytokines (Zhang et al., 2018). Edible nanoparticles with dendritic cells (DCs) tolerogenicity can prevent DCs activation via the AMPK pathway, promoting tolerance and protecting against colitis in mouse models (Deng et al., 2017). Furthermore, nanoparticles can be engineered to carry immunomodulatory molecules that modulate immune cells behavior. In the context of IBD, which is closely linked to immune disorders and excessive M1 macrophage activation, the M2-polarizing effect of interleukin-4 (IL-4) plays a crucial therapeutic role (Egawa et al., 2013; Risser et al., 2023). To harness this potential, a multilayered nanoarmor delivery system has been developed, designed to preserve the bioactivity of IL-4 and enable its targeted release within the inflammatory microenvironment. This system promotes M2 macrophage polarization, attenuating inflammation and promoting colitis tissue repair (Ge et al., 2023). In addition to directly targeting immune cells, nanoparticles can be engineered to modulate specific inflammatory pathways. For instance, a pH-responsive hydrogel-coated nanoemulsion was developed to co-deliver curcumin and emodin for targeted IBD treatment. This system effectively controls drug release in the colon, reduces inflammation, and promotes mucosal repair by decreasing pro-inflammatory factors (TNF-α and IL-6) and increasing anti-inflammatory factor (IL-10) through the NF-κB signaling pathway (Lei et al., 2023). Additionally, nanoparticles have shown significant promise in targeting the NLRP3 inflammasome, an intracellular complex that drives the release of pro-inflammatory cytokines such as IL-1β and IL-18 (Xu and Nunez, 2023), marking it as an emerging target in IBD therapy. Nanoparticles designed to inhibit NLRP3 activation effectively prevent the release of inflammatory mediators, offering a novel approach to controlling inflammation (Cai et al., 2021). In summary, nanomedicine leverages advanced delivery systems to target immune regulation in IBD, with a focus on macrophages and critical inflammatory pathways such as TNF-α, NF-κB, and NLRP3 inflammasome, providing a multifaceted and targeted strategy for IBD therapy.

Targeted nanomedicine holds promise in modulating immune responses in IBD, it also offers innovative strategies for addressing oxidative stress. Oxidative stress is another crucial aspect of the disease pathology (Geertsema et al., 2023), where an imbalance between ROS production and the antioxidant defenses of the body leads to lipid peroxidation, protein structure and function disruption, DNA damage, and ultimately, irreversible cell death (van der Pol et al., 2019; Georgiou and Margaritis, 2021). Nanoparticles can be employed to delivery antioxidants directly to specific areas, protecting them from degradation, enhancing their stability, and effectively reducing inflammation while promoting colonic tissue repair (Xu J. et al., 2022). A particularly innovative approach involves the development of ROS-responsive nanoparticle systems that release encapsulated drugs precisely in response to highly expressed ROS at diseased sites (Li et al., 2019; Xiao et al., 2021). Xudong Tang and his team developed ROS-responsive nanoparticles carrying the Fc-fused PD-L1 (programmed cell death-ligand) to enhance the targeted drug delivery and efficacy of PD-L1 pathway-based therapies for IBD (Tang et al., 2024). This approach ensures therapeutic agents to be released only where they are needed most, minimizing potential side effects and maximizing therapeutic efficacy. Moreover, synthetic nanoparticles, such as transition metal halides nano flakes (Guo et al., 2022) and genetically engineered probiotics (Zhou J. et al., 2022), mimic multi-enzyme activities, possessing antioxidant properties including peroxidase, superoxide dismutase (SOD), glutathione peroxidase (GPx), and catalase (CAT). They can be designed to specifically target inflamed colon tissues through electrostatic interactions, enhancing their adhesion to the affected areas (Min et al., 2023; Zhao et al., 2020). By scavenging ROS, these multi-enzyme nanoparticles not only mitigate oxidative damage but also contribute to restoring the intestinal barrier (Zhang X. et al., 2023), thereby promoting tissue repair and improving overall outcomes in IBD management. Targeted nanomedicine provides innovative strategies for combating oxidative stress in IBD. ROS-responsive nanoparticles enhance the delivery of antioxidants, reduce oxidative damage, and promote tissue repair, ultimately improving therapeutic outcomes in IBD treatment.

The integration of gut microbiota and nanotechnology also represents a state-of-the-art approach in the treatment of gastrointestinal diseases, particularly IBD. The gut microbiota plays a critical role in maintaining intestinal homeostasis, and its disruption is strongly linked to the pathogenesis of IBD (Schirmer et al., 2019; Qiu et al., 2022). Leveraging the unique properties of nanoparticles alongside the beneficial effects of gut microbiota can significantly enhance therapeutic outcomes. Food-derived nanoparticles can be absorbed by gut microbiota, allowing them to modulate the microbiome and host physiology through microRNA delivery. This approach addresses dysbiosis and alleviates IBD by influencing both the microbial community and the inflammatory responses (Teng et al., 2018). Additionally, polymer-based nanoformulations for oral administration have been shown to positively regulate gut microbiota, increasing the prevalence of probiotics while inhibiting pathogenic bacteria (Bao et al., 2023; Shah et al., 2020). Probiotics, such as Bifidobacterium bifidum and *Lactobacillus* acidophilus (Derakhshan-Sefidi et al., 2024), recognized for their potential to restore gut homeostasis, are emerging as promising candidates for IBD management (Roy and Dhaneshwar, 2023). However, their effectiveness is often limited by issues of stability and targeted delivery (Lopes et al., 2023). Encapsulation of probiotics within nanoparticles made from organic materials offers a potential solution to these challenges, protecting them from the harsh conditions of the gastrointestinal tract and ensuring that sufficient amounts are delivered directly to the colon (Li et al., 2023; Qiu et al., 2023). Genetically engineered probiotics offer a promising approach for IBD treatment by targeting immune regulation and modulating the gut microbiota. These probiotics can be genetically designed to express immune-modulating factors, enabling them to regulate immune responses and promote immune tolerance (Li et al., 2024). This strategy not only improves immune function but also helps restore microbial balance, offering a dual mechanism for alleviating IBD symptoms and promoting gut healing. Probiotic-derived extracellular vesicles have the potential to regulate immune responses, enhance intestinal barrier function, and modulate the gut microbiota (Zheng et al., 2023). However, this area of research is still in its early stages, with much to be explored regarding the mechanisms and effectiveness in IBD treatment. This synergy between nanotechnology and gut microbiota not only improves the effectiveness of treatment but also minimizes drug loss, offering a cutting-edge approach to IBD treatment. Although the application of genetically engineered probiotics and probiotic-derived extracellular vesicles is still in its early stages, their promising potential opens new possibilities for IBD treatment.

In addition to targeting the underlying pathogenic mechanisms of IBD, advancements in nanomedicine have led to the development of innovative nanomaterials that offer enhanced advantages. Currently, nanomaterials utilized in IBD treatment can be broadly categorized into synthetic nanoparticles, lipid-based nanoparticles (LNPs), metal and metal-oxide nanoparticles, and extracellular vesicles. Synthetic nanoparticles, such as polymer-based nanoparticles and nano-hydrogel composites, have long been a cornerstone of nanomedicine due to their design versatility, controlled release capabilities, and ability to encapsulate a wide variety of therapeutic agents (Ahlawat et al., 2018). Advances in synthetic nanoparticle engineering, including the development of biomimetic polydopamine nanoparticles (Bao et al., 2023) and biodegradable poly lactic-co-glycolic acid nanoparticles (Puricelli et al., 2023), have significantly enhanced the bioavailability and therapeutic efficacy of drugs in IBD by improving their biocompatibility while maintaining solubility and stability (Adabi et al., 2017; Zor et al., 2019). LNPs have gained significant prominence following their success in mRNA vaccines development (Cullis and Felgner, 2024). LNPs provided a versatile platform for nucleic acid delivery by overcoming challenges in nucleic acid degradation and limited cellular uptake (Hald Albertsen et al., 2022). Sung et al. demonstrated the potential of LNPs encapsulating IL-22-mRNA to specifically target injured intestinal mucosa and effectively treat ulcerative colitis (Sung et al., 2022). This highlights their promise as an emerging tool in IBD therapy. Metal and metal-oxide nanoparticles, while less emphasized due to potential toxicity concern (Bi et al., 2023), hold significant value in IBD therapy for their antioxidant properties. Additionally, they serve as contrast agents in imaging and diagnostics, offering dual benefits of therapeutic and diagnostic capabilities (Ando et al., 2013). Biological-derived nanovesicles, as naturally targeted drug delivery systems, have emerged as a cutting-edge approach in the treatment of IBD. These membrane-bound nanoparticles are capable of carrying a variety of bioactive molecules, including RNAs, proteins, and lipids, allowing them to restore gut barrier function effectively (Eom et al., 2022). Studies have highlighted the potential of nanovesicles derived from plant and probiotics, such as turmeric (Gao et al., 2022), aloe (Choi et al., 2023), garlic (Zhu et al., 2023), and *Akkermansia muciniphila* (Zheng et al., 2023) in promoting gut permeability and enhancing intestinal barrier function primarily by regulating the balance of gut microbiota. These nanovesicles also relief IBD symptoms through additional mechanisms. For instance, turmeric-derived exosome-like nanovesicles have demonstrated potent anti-inflammatory and antioxidant properties in IBD models, accumulating in inflamed colonic sites and reducing pro-inflammatory cytokine levels, thereby aiding in the repair of the gut barrier function (Liu C. et al., 2022). Moreover, exosomes derived from mesenchymal stem cells (MSC-Exos) have exhibited significant anti-inflammatory and immunomodulatory activities, specifically targeting inflamed tissues and enhancing the repair of damaged tissues (Tian et al., 2023). The emergence of biomimetic nanovesicles, such as specialized leukosomes (Corbo et al., 2017) and nanovesicles expressing OX40 receptors (Fu et al., 2021), underscores the potential of engineered nanovesicles to enhance treatment specificity and reduce side effects (Mougenot et al., 2022). Taken together, these innovations, as both targeted drug delivery systems and nano-therapeutics, offer a more effective and safer strategy for managing IBD.

Furthermore, utilizing nanoparticles within a single delivery system offers a multifunctional approach that simultaneously targets various pathological mechanisms of IBD, leading to a more comprehensive treatment strategy (Zhang X. et al., 2023; Kang et al., 2024). Dr. Tinnirello and his team developed industrially produced lemon nanovesicles that exhibited both anti-inflammatory and antioxidant effects. They not only reduce inflammatory markers by modulating the NF-κB and Nrf2 pathways but also restore gut microbial balance, effectively ameliorating 2,4 dinitrobenzensulfuric acid (DNBS)-induced colitis (Tinnirello et al., 2024). This synergistic effect enhances overall therapeutic efficacy by targeting multiple pathways involved in the disease processes, providing a more effective and nuanced method for managing IBD.

In conclusion, emerging hotspots and frontiers in nanomedicine applications for IBD mainly focus on several key aspects: Firstly, the development of nanodelivery systems has transformed treatment approaches by enabling precise targeting and controlled release of therapeutic agents directly at inflamed sites. Secondly, nanoparticles are now being designed to target various pathological mechanisms of IBD, including immunomodulation by targeting specific immune cells to regulate inflammatory pathways, strategies to combat oxidative damage leveraging ROS-responsive nanoparticles to reduce oxidative stress, and the incorporation of gut microbial homeostasis by using nanoparticles that modulate gut microbiota. These three areas represent critical mechanisms underlying the pathogenesis and progression of IBD, and nanotechnology offers unique advantages in precisely targeting these pathways to enhance therapeutic outcomes. Lastly, biological-derived nanovesicles, which carry diverse bioactive molecules and aid in repairing intestinal barrier function, also are effective in relieving IBD symptoms. Collectively, these innovations represent a multifunctional approach that addresses multiple pathological mechanisms of IBD, offering a more comprehensive and effective treatment strategy.

### 4.3 Strengths and limitations

Overall, this study presents the first bibliometric analysis of nanomedicine applications in IBD-related publications over the past two decades. It covers trends in publications, international collaboration, and research hotspots, providing the scientific community with a fresh and objective overview of evolving research topics that are poised to drive future nanomedicine applications in IBD. As part of the research, a multidimensional analysis was conducted simultaneously using various bibliometric software tools, particularly VOSviewer and CiteSpace, which are widely recognized in the field of bibliometrics. These approaches allow our analysis to offer a more comprehensive insight into the hotspots and emerging frontiers, providing readers perspectives that extend beyond what can be achieved through traditional reviews. Of course, this study has some limitations. Firstly, the data were sourced solely from the WoSCC database, which means that relevant studies from PubMed, Scopus, and other databases may have been excluded, potentially omitting some pertinent research. However, it should be noted that WoSCC indexes the largest number of scholar publications, ensuring the integrity of source data (Jin et al., 2023). Secondly, by filtering for studies published only in English, there is a possibility that high-quality articles in other languages may have been underestimated. Finally, due to methodological limitations in the literature quality evaluation system, some newly published high-quality documents with low citations and centrality might not have been included in the bibliometrics analysis.

## 5 Conclusion

This bibliometric analysis delves into the evolution, trends, and emerging frontiers of nanomedicine applications in IBD over the past two decades, revealing the rapid growth and increasing complexity of this interdisciplinary field. The findings underscore the significant contributions from leading institutions and researchers globally, with China and the United States at the forefront of research output and influence. However, the analysis also highlights the need for enhanced collaboration among authors. A strong foundation in fundamental sciences, particularly chemistry and materials science, have paved the way for growing clinical applications of nanomedicine in IBD. The development of advanced nanodelivery systems, immunomodulatory approaches, strategies to mitigate oxidative damage, innovations in gut microbial homeostasis, and biological-derived nanovesicles have emerged as key hotspots and trends shaping the future of this field. Our study provides a comprehensive framework for understanding the current landscape of nanomedicine applications in IBD, suggesting that a dynamic field is poised for continued growth. Future research is likely to focus on further enhancing the precision, efficacy, and safety of nanomedicine applications, ultimately advancing the treatment of IBD.

## Data Availability

The raw data supporting the conclusions of this article will be made available by the authors, without undue reservation.
